# Implications for a Stem Cell Regenerative Medicine Based Approach to Human Intervertebral Disk Degeneration

**DOI:** 10.3389/fcell.2017.00017

**Published:** 2017-03-07

**Authors:** Petra Kraus, Thomas Lufkin

**Affiliations:** Department of Biology, Clarkson UniversityPotsdam, NY, USA

**Keywords:** stem cell, pluripotency, regenerative medicine, differentiation, intervertebral disc, lower back pain, mesenchymal stem cells, ips cells

## Abstract

The human body develops from a single cell, the zygote, the product of the maternal oocyte and the paternal spermatozoon. That 1-cell zygote embryo will divide and eventually grow into an adult human which is comprised of ~3.7 × 10^13^ cells. The tens of trillions of cells in the adult human can be classified into approximately 200 different highly specialized cell types that make up all of the different tissues and organs of the human body. Regenerative medicine aims to replace or restore dysfunctional cells, tissues and organs with fully functional ones. One area receiving attention is regeneration of the intervertebral discs (IVDs), which are located between the vertebrae and function to give flexibility and support load to the spine. Degenerated discs are a major cause of lower back pain. Different stem cell based regenerative medicine approaches to cure disc degeneration are now available, including using autologous mesenchymal stem cells (MSCs), induced pluripotent stem cells (iPSCs) and even attempts at direct transdifferentiation of somatic cells. Here we discuss some of the recent advances, successes, drawbacks, and the failures of the above-mentioned approaches.

## Human stem cell totipotency

The most stringent definition of human totipotency is defined as the ability of a single cell when placed in its normal environment (i.e., uterus) to undergo a developmental program that eventually gives rise to an offspring which itself is fertile (Condic, 2014). In human embryos experimental evidence indicates that the cells of the embryo are totipotent from the 1-cell stage (zygote) up until the 4-cell stage, where it has been shown that at least one of the blastomeres of the 4-cell embryo (after reimplantation into the uterus) is capable of giving rise to a fertile human (Veiga et al., 1987; Van De Velde et al., 2008; De Paepe et al., 2014). It has been suggested that the blastomeres of the 8-cell embryo are also totipotent as individual blastomeres are able to give rise to both the inner cell mass (ICM) and trophectoderm (TE) of the blastocyst (Mottla et al., 1995). Furthermore, molecular studies have shown that the transcriptomes of individual blastomeres taken from 8-cell embryos are essentially identical, suggesting that they have yet to make any molecular decisions regarding lineage commitment or differentiation (Galan et al., 2010). But using the strict definition of totipotency as described above, evidence is lacking that the individual blastomeres of the 8-cell human embryo are indeed totipotent.

## Pluripotency

It has been estimated that the human body is made up of ~3.7 × 10^13^ cells (Bianconi et al., 2013) and by histological analysis these cells are commonly divided into ~200 different cells types (reviewed in Alberts et al., 2015). The developmental potency of *in vitro* established human cell lines isolated from preimplantation stage human embryos has been studied intensively over the past several years. The earliest developmental stage of human embryonic stem cells (hESCs) isolated *in vitro* are referred to as “naïve” (meaning of greater developmental potential) and subsequently transitions through to a stage of lesser potential termed “primed” (reviewed in Nichols and Smith, 2009; Davidson et al., 2015; Ávila-González et al., 2016; Hockemeyer and Jaenisch, 2016; Weinberger et al., 2016; Wu et al., 2016 and references therein). Cells of the primed stage are equivalent to epiblast cells of the preimplantation human embryo. Epiblast cells are capable of giving rise to the each of the three principal germ layers, ectoderm, mesoderm, and endoderm of the embryo. The mesodermal layer is what gives rise to the notochord and somites, the two precursors which eventually give rise to the cells of the nucleus pulposus (NP) and annulus fibrosus (AF), respectively, of the intervertebral disc (reviewed in Sivakamasundari and Lufkin, 2012, 2013).

## Multipotent mesenchymal stem cell

Mesenchymal stem cells (MSCs) are termed “multipotent” as they were initially shown to be able to differentiate into three different lineages, namely adipocytes, osteoblasts, and chondrocytes (Pittenger et al., 1999). MSCs were initially found in adipose tissue, dental pulp, bone marrow, and circulating blood. Recent advances with MSCs have shown they also have the ability to differentiate into myocytes, cardiomyocytes, fibroblasts, myofibroblasts, epithelial cells, and neurons (reviewed in Liu et al., 2009) and can be found in other hematopoietic sites such as fetal cord blood and liver. Recent studies indicate an even greater number of tissues which contain MSCs (Perez-Silos et al., 2016). Given the larger number of cell types which the MSCs are capable of differentiating into, and the different germ layers from which these differentiated cells are normally derived, it might be more appropriate to consider them pluripotent rather than only multipotent. Additionally MSCs are phenotypically quite heterogeneous, so current criteria for what constitutes a MSC is that it must: (1) adhere to plastic cell culture dishes; (2) express certain CD markers in the majority (95%) of the population (CD105, CD90 and CD73) and not express a group of six CD markers as well as HLA-DR in the same population and; (3) be able to differentiate into adipocytes, osteoblasts and chondrocytes (reviewed in Liu et al., 2009).

## Why is the intervertebral disc important?

**Figure 1:**
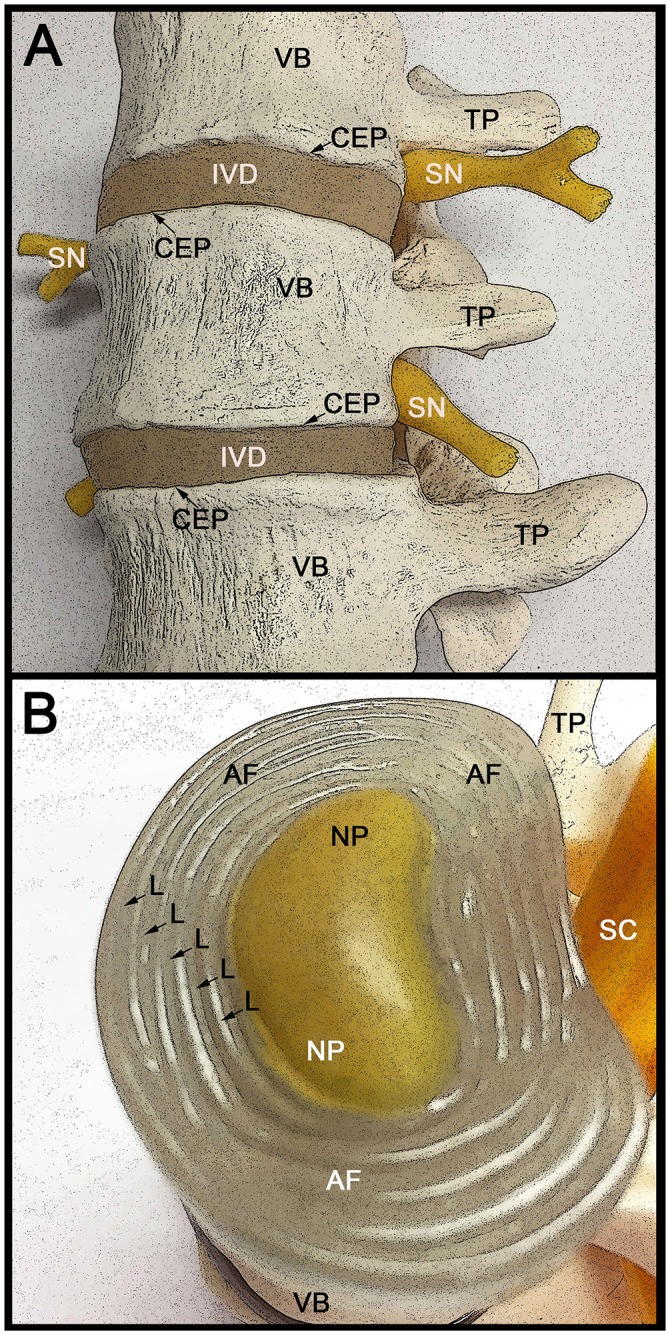
**Anatomical structure of the lumbar spine and intervertebral discs. (A)** Lateral view of three adjacent human vertebrae (VB) with two intervening intervertebral discs (IVD). The VB attaches to the IVD through the cartilage end plates (CEP). The spinal nerve (SN) roots exiting the spinal cord are indicated, as well as the transvers processes (TP) that connect the VB to the spinous process (not visible). With increased age the IVD becomes thinner in part due to fluid loss from the extracellular spaces. This can result in the formation of bone spurs (osteophytes), which in turn can impinge on the spinal nerve roots causing severe pain. **(B)** Rostral (superior) view of the intervertebral disc (IVD) with the CEP removed to allow better visualization of the internal IVD. The concentric circles of lamellae (L) that make up the annulus fibrosus (AF) are indicated with arrows. The centrally located gelatinous nucleus pulposus (NP) is indicated and is surrounded by the AF. The caudally (inferior) located vertebral body (VB) and transverse process (TP) are indicated, as well as the location of the spinal cord (SC).

The vertebral column is the most critical physical support framework for vertebrates, functioning to provide mechanical support along with flexibility, as well as protection of the spinal cord and associated spinal nerves (Figure 1). The vertebral column is comprised of a characteristic metameric arrangement of the vertebral bodies linked together by intervertebral discs (reviewed in Sivakamasundari and Lufkin, 2012, 2013; Lawson and Harfe, 2015). An indispensable aspect of the vertebral design, the intervertebral disc (IVD) serves to withstand biomechanical forces and confers tensile strength and flexibility in motion to an otherwise rigid spine (Risbud and Shapiro, 2011). Intervertebral disc degeneration is a prevalent spinal malady, which can lead to back pain, as can more severe spine afflictions like spinal stenosis, IVD herniation, or radiculopathy. At some point in their lives, over 75% of the population will be affected by low back pain (Raj, 2008). In the USA the annual expenditures related to chronic back pain can surpass $35 billion in terms of medical health care costs and the reduction in productivity from lost workdays. This exceeds the combined costs of coronary artery disease, stroke, rheumatoid disease, diabetes, and respiratory infection and imposes an enormous socio-economic burden (Walker, 2000; Goetzel et al., 2003; Katz, 2006).

## Developmental biology of the intervertebral disc

The IVD is an unique non-vascularized fibrocartilage tissue consisting of a lamellar-structured circular AF surrounding the central NP core and incased above (superior) and below (inferior) by the vascularized cartilage endplates through which nutrients can diffuse to the AF and NP (Sivakamasundari and Lufkin, 2012; Kraus and Lufkin, 2016). The IVD is the largest non-vascularized tissue in the body. The IVD tripartite structure functions in an integrated biophysical manner, with the hydrogel-like NP acting to resist compression of the spine, while the fibrous design of the AF permits it to resist tension and simultaneously retain and support the NP during spinal loading. The endplates function to hydraulically encase the NP and AF above and below, as well as providing limited access to nutrients and structural linkage to the adjacent vertebral bodies (Sivakamasundari and Lufkin, 2013). Molecular genetic analysis in mice indicates that the NP is derived from the notochord, a structure that is laid down by the retreating node along the rostrocaudal axis during gastrulation (reviewed in Lawson and Harfe, 2015). In contrast, the AF, cartilage endplates, and vertebral bodies are all derived from the sclerotome portion of the somite (Sivakamasundari et al., 2017). The forkhead-related protein transcription factors Foxa1 and Foxa2 appear to play a critical role in the transition of notochordal cells to NP cells, as in their absence the NP fails to form (Maier et al., 2013). Likewise, the paired-domain containing transcription factors Pax1 and Pax9 are critical for formation of the AF from the sclerotome, where it has been shown that they are connected to the chondrogenic transcription factors Sox5 and Sox6 through a negative feedback loop. Pax1 and Pax9 appear to mediated their effect in part via modulation of the BMP and TGF-B pathways (Sivakamasundari et al., 2017).

## Treating IVD disease with MSCs

Mesenchymal stem cells (MSCs) have been studied as a potential source material for treating cartilaginous skeletogenic defects, and with regards to the IVD the majority have focused on NP cells (reviewed in Sivakamasundari and Lufkin, 2012, 2013). The bulk of these studies resulted in cells that were closer in phenotype to chondrocytes, rather than NP cells. Chondrogenic differentiated MSCs are unlikely to be the best choice for repair of a damaged disc. Injection of such cells in the NP does not result in the appropriate hydrogel matrix characteristic of the NP, but instead results in the generation of hyaline cartilage. Notochordal cells have been investigated as a substrate to coax MSCs toward an NP phenotype (Purmessur et al., 2011). Micromass cultured human MSCs when exposed to notochord cell conditioned medium showed an increase in glycosaminoglycan (GAG) and an up-regulation of SOX9 and COLII gene expression (Purmessur et al., 2011) hence the identification and optimization of the soluble factors produced by notochordal cells could provide a supporting strategy for directing MSCs to a NP phenotype. Thus being able to coax MSCs toward an AF or NP fate will be crucial to ensure future effective disc repair. Progress in this direction has been hampered in part by a poor consensus on identifying markers which clearly distinguish between NP and AF cells (Thorpe et al., 2016). In fact some studies suggest that both the NP and AF may be composed of a heterogeneous cell population (Van Den Akker et al., 2014, 2016; Thorpe et al., 2016; Kraus et al., 2017). Nevertheless, such studies have served as a jumping off spot to adjust the *in vitro* cell culture conditions to more directly obtain AF or NP cells from multipotent stem cells (Hofer and Tuan, 2016).

## Directing stem cells toward an NP or AF lineage

The majority of studies on differentiating mesenchymal stem cells (MSCs) to an IVD cell type have focused on the NP cell phenotype. In contrast, AF cells have received less attention, although repair of the AF would be of great utility in reversing the effects of herniations and tears which result in prolapsed or herniated “slipped” discs. Recent animal studies suggest that BMSCs could be an effective treatment to a damaged AF (Li et al., 2016). The AF is comprised of water and an intricate concentric layering of crisscross oriented elastic collagen fibers known as lamellae. The crisscross orientation of the lamellae give the AF greater strength not unlike the concentric belts in an automobile tire. The AF can be subdivided into two parts, the inner AF (IAF) and the outer AF (OAF). The IAF is cartilaginous during embryogenesis and expresses large amounts of Collagen II, whereas the OAF is more fibrous and expresses a greater amount of Collagen I (Sivakamasundari et al., 2017). This demarcation between IAF and OAF become less pronounced at later stages of development and in the adult. (Hayes and Ralphs, 2011). The fact that the entire spine research field has so far been unsuccessful in generating true AF and NP cells from MSCs suggests that they might not be the ideal starting material despite their relative ease of isolation and autologous nature. Their stem cell potency as described above may be limited such that they cannot adopt either the AF or NP identity or important clues from the extracellular matrix are missing. It is for that reason that work with hESCs remains important, and resulting differentiation protocols are likely transferable to autologous induced pluripotent stem cells (iPSCs).

## Successful stem cell trials in animals

Animal studies suggest that the IVD contains a resident stem cell population (Shi et al., 2015; Kraus et al., 2017), however this work was performed in rodents and bovine so it is unclear if the same cell populations will be found in humans. Interestingly, the canine breed Dachshund is commonly afflicted with disc degeneration and disc herniation in a manner similar to humans (Hoffman and Dow, 2016). Like humans, afflicted canines can show back pain, impaired movement, or near paralysis. In several studies using mesenchymal stem cells injected into affected discs, treated canines showed improvement, including reduced pain, ataxia, improved reflexes, and neurologic-locomotory recovery although surprisingly there did not appear to be any ultrastructural changes as monitored by MRI (reviewed in Hoffman and Dow, 2016). This underscores the utility of using an animal model (canine or bovine Kraus et al., 2017) which are more similar to human and also indicates that the criteria for stem cell mediated clinical success of disc disease might be focused on alleviation of symptoms and recovery of normal activity rather than ultrastructural changes in the spine as assayed by MRI.

## Stem cells in human clinical trials for IVD disease

In a recent controlled study of 24 patients with chronic low back pain, 12 received a transplantation of allogeneic mesenchymal bone marrow cells via intradiscal injection (Noriega et al., 2016). Five of the treated patients (~40%) displayed a quick and significant improvement in pain relief suggesting that this simple, non-surgical procedure can provide pain relief to a certain percentage of those suffering from chronic low back pain (Noriega et al., 2016). In another recent independent study on humans (Elabd et al., 2016), five patients who presented with degenerated discs had bone marrow mesenchymal stem cells removed and cultured *in vitro* under hypoxic conditions, which is the normal state of the IVD. The patients subsequently received intra-discal injections of their own (autologous) hypoxic cultured cells. Follow up studies after several years including MRI showed no aberrant growths (tumors) or abnormalities and the patients reported improvements in both mobility and strength (Elabd et al., 2016). These results suggest the feasibility and safety of this approach. Future work should possibly focus on a randomized, controlled, double blind study with a larger number of patients.

The U.S. National Institutes of Health has a searchable database for clinical trials (https://clinicaltrials.gov). At the time of this writing a search identified 36 studies that have been registered for stem cell treatments of defective or diseased intervertebral discs. Eight of these studies are currently recruiting patients. One ongoing trial with an estimated enrollment of 360 patients is being directed by Mesoblast, Ltd (Mesoblast, 2016). The company which is based in Melbourne, Australia and New York, NY features proprietary allogeneic mesenchymal adult stem cells (MLCs) which are purported to be found adjacent to blood vessels. Using monoclonal antibodies they are able to isolate from them mesenchymal precursor cells (MPCs) that are subsequently expanded in culture without differentiation. Apparently MSCs can be derived from MPCs in cell culture following density gradient separation and adherence to tissue culture plastic ware, a characteristic of stem cells. One line of MPCs is referred to as Rexlemestrocel-L and it will be used in the clinical trial (Mesoblast, 2016) which has a projected completion date in 2020. Based upon the above-described successes with intra-discal injections of MSCs, one could hope for a favorable outcome.

## Will iPSCs play a significant role in IVD regeneration

Induced pluripotent stem cells (iPSCs) either derived from the patient needing treatment (autologous) or obtained from a cell bank using a human leukocyte antigen (HLA) matched line both hold great promise as approaches in regenerative medicine. However, it appears that the former strategy is very time consuming and quite costly as extensive quality control (such as entire genome sequencing) needs to be performed on each of the individual patient-specific iPSC lines owing to the elevated risk of generating random mutations, which could possibly lead to tumor formation during the iPSC generation protocol (Kamao et al., 2014). Homozygous HLA matched iPSC quality tested, banked and clinically safe cell lines from healthy donors (like those found at the CiRA Center, Kyoto University, Japan) also hold much promise for stem cell based therapies (Sugita et al., 2016; Takahashi and Yamanaka, 2016), but the current limited number of lines available makes this an option for only a small percent of the world's population (Karagiannis and Eto, 2016; Sakurai et al., 2016). In a recent interview, Shinya Yamanaka, who developed the strategy for iPSC generation (Takahashi and Yamanaka, 2006, 2016) suggested that iPSCs would likely only be useful for treating approximately nine human diseases (spinal cord injury, joint disorders, Parkinson's, specific blood disorders, heart and liver failure, retinal and corneal diseases, and diabetes) over the next decade or so, and IVD-related diseases were not among them (Ravven, 2017). That is not to say that at some point in the future iPSCs will not be utilized to treat degenerative disc diseases or other IVD-related pathologies, but rather it does not appear to be on the immediate horizon.

## Conclusion

Important advances in regenerative medicine are at our doorsteps for the treatment of degenerative diseases that emerge with an increasingly longer life span, eventually enabling safe replacement of failing body parts, such as degenerated IVDs. All cells of a mature organism have once shared the totipotent origin of the 1-cell zygote. MSCs are just one type of pluripotency restricted differentiation product thereof generated through the concerted interaction of complex transcription factor networks during development. While the numbers of clinics and private practices offering MSC-based stem cell treatments for various degenerative illnesses, including degenerative disc disease, are on the rise, it remains important to understand the precise fate and contribution of these cells following their use in a therapeutic context and as such, promote a safer outcome for regenerative medicine based approaches to degenerative diseases.

## Author contributions

TL and PK were involved in the writing of this mini-review inaugural article.

## Funding

This work was funded by the Bayard and Virginia Clarkson Endowed Chair in Biology to TL.

### Conflict of interest statement

The authors declare that the research was conducted in the absence of any commercial or financial relationships that could be construed as a potential conflict of interest.
